# Clinical Impact of Consolidative and Salvage Radiotherapy for Lymph Node Metastasis in Upper Urinary Tract Urothelial Carcinoma

**DOI:** 10.1155/2018/1471839

**Published:** 2018-04-22

**Authors:** Hideyuki Kondo, Suguru Shirotake, Takashi Okabe, Soichi Makino, Koshiro Nishimoto, Masafumi Oyama

**Affiliations:** Department of Uro-Oncology, Saitama Medical University International Medical Center, Saitama, Japan

## Abstract

A 75-year-old Japanese male was referred to our institution for the evaluation of a left ureteral tumor in the ureterovesical junction. Computed tomography and pathologic examination under ureteroscopy revealed an invasive left ureteral urothelial carcinoma with left obturator nodal metastasis without distant metastasis. First, the patient underwent systemic chemotherapy (gemcitabine and cisplatin chemotherapy). We then performed left radical nephroureterectomy and extended lymph node dissection. Pathological examination revealed that the tumor was a high-grade invasive urothelial carcinoma with left common iliac and pelvic lymph node metastasis (pT3N2). Unfortunately, metastases appeared in the common iliac and para-aortic lymph nodes immediately after the operation; therefore, the previous first-line chemotherapy was readministered and second-line chemotherapy (gemcitabine and paclitaxel chemotherapy) was also performed. We also performed consolidative radiotherapy and salvage radiotherapy (boost, 20 Gy/10 fractions to the inferior para-aortic, and left common iliac regions containing swollen lymph nodes). The patient has shown no evidence of recurrence or metastasis even approximately 4 years after the initial diagnosis of advanced UUT-UC with lymph node metastasis. Our case suggests that consolidative or salvage radiotherapy combined with surgery and chemotherapy may provide clinical benefit for selected cases of advanced UUT-UC with lymph node metastasis.

## 1. Introduction

Upper urinary tract urothelial carcinoma (UUT-UC) is relatively rare and accounts for 5–10% of urothelial malignancies [1]. Generally, the 5-year survival rates of patients with pTa or pT1 UUT-UC (Stage 0-I in the AJCC staging system) are over 60%, whereas the rate for patients with pT3-4 or N1-3 disease (Stage IV) is extremely poor (<5%) [2]. According to a report of a large series of patients with UUT-UC treated with radical nephroureterectomy (RNU), the 5-year recurrence-free survival rates of pT4 and N1-3 diseases were only 4.7% and 29%, respectively, and therefore the application of multimodality therapy for Stage IV UUT-UC disease may be considered. Herein, we report a case of Stage IV UUT-UC that was successfully treated with RNU, chemotherapy, and radiotherapy.

## 2. Case Description

A 75-year-old Japanese male, who was admitted to another hospital with renal dysfunction and microscopic hematuria at a medical checkup, was diagnosed with a left lower ureteral tumor. The patient was referred to the Saitama Medical University International Medical Center for treatment of the tumor. As for the relevant past history, the patient had received stent-graft implantation for the treatment of abdominal aortic aneurism. There was no significant family history. Physical examination revealed normal appearance except for high body mass index (27.3 kg/m^2^ [normal range < 25 kg/m^2^], body weight: 78.4 kg, height: 1.69 m). Laboratory data were normal except for serum creatinine (1.56 [normal range: 0.43–1.08] mg/dl) and creatinine clearance (50.2 ml/min). Urine cytological examination detected urothelial carcinoma cells. Magnetic resonance imaging suggested the ureteral tumor (21 × 13 mm) had invaded the muscle layer, but not the periureteric fat (Figure S1). Computed tomography (CT) showed a contrast-enhanced tumor in the lower ureter (data not shown), left hydronephrosis (data not shown) and an enlarged left obturator lymph node (18 mm in diameter, Figure S2). There was no significant finding in the bladder using cystoscopy. By tumor biopsy using a ureteroscope, the tumor was pathologically diagnosed as a high-grade urothelial carcinoma (UC, clinical stage: cT2, N1, M0). The patient underwent several modality therapies (Table S1). The treatment efficacy was evaluated by CT imaging (Figure S2), in which the sizes of the LNs were retrospectively measured by HK, SS, TO, and AK (Table S1) and compared by statistical analysis (Student's *t*-test). In the comparative analysis, a *p* value below 0.05 was considered significant.

**Figure 1:**
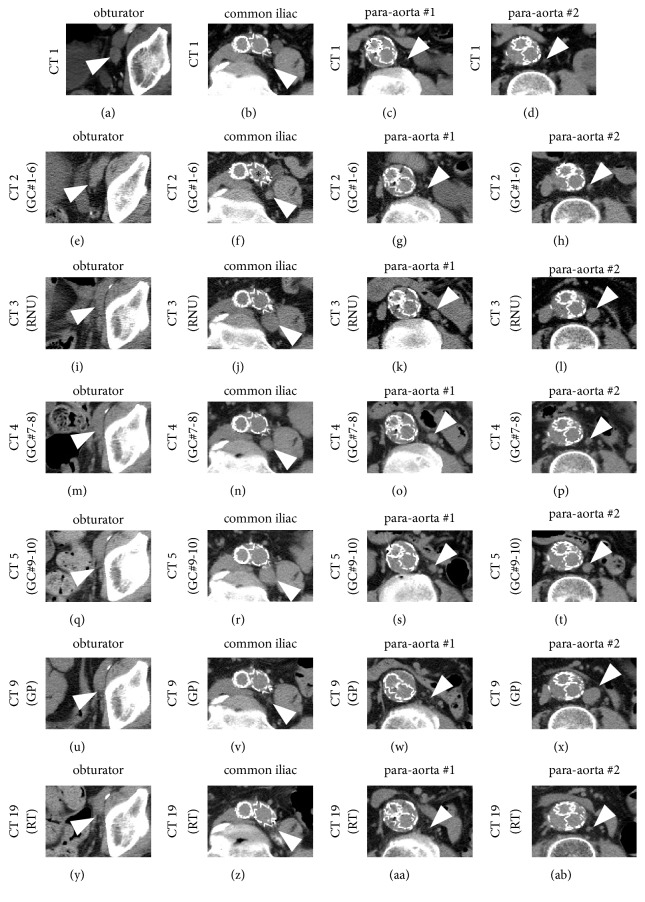
*Selected CT images of the lymph nodes for every treatment modality*. (a–d), (e–h), (i–l), (m–p), (q–t), (u–x), and (y–ab) represent CT scans #1, #2, #3, #4, #5, #9, and #19 in supplemental Figure 1, respectively. Arrowheads in the 1st to 4th columns show the obturator lymph nodes, common iliac lymph nodes, para-aortic lymph node #1, and para-aortic lymph node #2, respectively. CT: computed tomography, GC: chemotherapy using gemcitabine and cisplatin, RNU: radical nephroureterectomy, GP: chemotherapy using gemcitabine and paclitaxel, and RT: radiotherapy.

In order to control the apparent obturator LN metastasis (17.5 ± 0.5 mm, Figure 1(a), Table S1), the patient was given 6 courses of chemotherapy with gemcitabine (1,000 mg/m^2^, days 1, 8, and 15) and cisplatin (70 mg/m^2^, day 2) (GC-chemotherapy, GC #1-6) [3, 4]. Other LNs, which subsequently showed enlargement, were within normal size (<1.0 cm) before GC-chemotherapy (Figures 1(b)–1(d)). Although the obturator LN significantly shrunk to 8.4 ± 1.1 mm (Figure 1(e)), the left common iliac LN enlarged from 6.6 ± 0.4 to 11.0 ± 0.4 mm (Figure 1(f)). The two para-aortic LNs, which later expanded, remained within the normal size range (6.4 ± 0.3 and 7.1 ± 0.8 mm, resp., Figures 1(g)-1(h)).

In order to treat the ureteral tumor and common iliac LN metastasis, in which cancer cells may acquire resistance to GC-chemotherapy, the patient underwent left RNU and lymph node dissection (LND), including most of the common iliac LNs. Simultaneously, regional external iliac, obturator, and internal iliac LNs were removed as previously reported [5, 6]. However, we could not remove the enlarged LN behind the common iliac artery (*∗* in Figure 1(f)) owing to vascular adherence, probably caused by stent-graft implantation for the treatment of aortic aneurism (Figure 1(f)). The pathological diagnosis was a high-grade UC that had invaded the periureteric fat and metastasized to two of four LNs (pT3N2).

At 1.5 months after the surgery, CT revealed no recurrence to the obturator lymph node (Figure 1(i)), but the remaining common iliac LN enlarged to 22.2 ± 0.8 mm (Figure 1(j)). Furthermore, the sizes of the two para-aortic LNs significantly increased to 12.7 ± 0.9 mm (para-aortic LN #1, Figure 1(k)) and 17.1 ± 0.8 mm (para-aortic LN #2, Figure 1(l)). The patient underwent 2 additional courses of GC-chemotherapy (GC #7-8). The common iliac LN and para-aortic LN #2 shrank, to 15.5 ± 0.9 mm (Figure 1(n)) and 7.9 ± 0.6 mm (Figure 1(p)), respectively, but the para-aortic LN #1 did not (Figure 1(o)). After the additional 2 courses of GC-chemotherapy (GC #9-10), both obturator LN and para-aortic LN #1 remained within the normal size range (Figures 1(q) and 1(s)), whereas the sizes of the common iliac LN (23.0 ± 0.7 mm, Figure 1(r)) and para-aortic LN #2 (11.9 ± 0.4 mm, Figure 1(t)) increased.

The patient underwent 8 courses of second-line chemotherapy using gemcitabine (1,000 mg/m^2^, days 1, 8, and 15) and paclitaxel (180 mg/m^2^, day 1) (GP-chemotherapy #1-8, Table S1) [7]. No recurrence was observed at the site of the obturator LN resection (Figure 1(u)), while those of the common iliac LN (5.8 ± 0.7 mm, Figure 1(v)) and para-aortic LN #1 (5.8 ± 0.6 mm, Figure 1(w)) significantly decreased; however, the size of the para-aortic LN #2 grew (21.5 ± 0.3 mm, Figure 1(x)).

**Figure 2:**
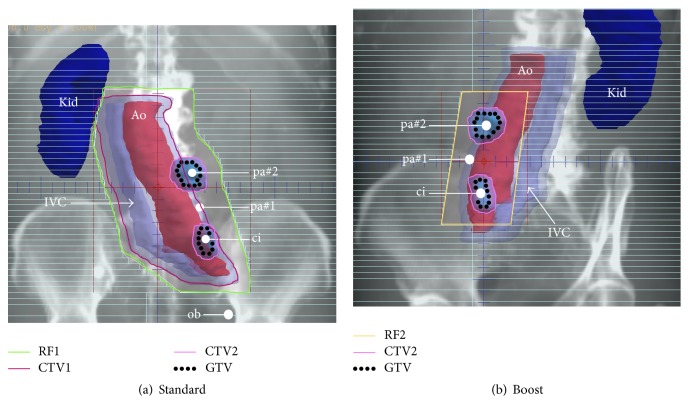
*Irradiation conditions for the lymph nodes*. (a) Frontal view of CT image reconstruction; RF1 was set based on CTV1 and 2 as standard radiation. (b) Left anterior oblique view of CT image reconstruction; RF2 was set based on CTV2 as a radiation boost. CT: computed tomography, RF: radiation field, GTV: gross tumor volume, CTV: clinical target volume, Ao: aorta, IVC: inferior vena cava, Kid: right kidney, ob: obturator lymph node, ci: left common iliac lymph node, and pa: para-aortic lymph node.

As consolidative therapy for the left common iliac LN and para-aortic LN #1 as well as salvage therapy for the para-aortic LN #2, the patient underwent aggressive extra-beam radiotherapy utilizing 10 megavolts photons from a linear accelerator employing a 3-dimensional conformal technique (Figure 2). The final irradiation dose was 60 gray (Gy) in 30 fractions. The patient underwent two patterns of radiotherapy: (A) standard irradiation from the frontal and posterior positions (40 Gy/20 fractions, green-lined area in Figure 2(a)) and (B) boost irradiation from the oblique position (20 Gy/10 fractions, yellow-lined area in Figure 2(b)). During standard irradiation, the gross tumor volume (GTV), identified on CT of the target metastasized lesions in the area, included the para-aortic LN #2 and the left common iliac LNs (para-aortic LN #2 and the left common iliac artery/vein, black-dotted lines in Figure 2(a)). Two clinical target volumes (CTVs, i.e., the clinical target of irradiation) were set to the overlapping areas (CTV1 and CTV2 in Figure 2(a)). CTV1 was defined by the aorta (Ao), inferior vena cava (IVC), and left common iliac artery/vein with 7 mm margins where metastatic cells possibly existed in the lymphatic system, while CTV2 was defined by the GTVs with a 5 mm margin (pink-circled area in Figure 2(a)). Subsequently, both actual radiation fields, RF1 and RF2, were set based on CTV1 and CTV2, excluding the right kidney, spinal cord, and bowel (green- and yellow-lined areas in Figures 2(a) and 2(b), resp.).

No recurrence was observed at the site of the extracted obturator LN (Figure 1(y)) with no regrowth reported in the two LNs that were irradiated as part of the consolidative therapy (Figures 1(z) and 1(aa)). Remarkably, the para-aortic LN #2 that presumably contained chemoresistant cancer cells significantly shrunk to normal size after salvage radiotherapy (5.4 ± 0.5 mm, Figure 1(ab)). The patient is currently free from cancer recurrence/metastasis and severe complications possibly due to aggressive multimodality therapies.

## 3. Discussion

We have described here a case of Stage IV UUT-UC with LN metastases successfully treated with multimodality therapies including (i) RNU with LND after neoadjuvant GC-chemotherapy, (ii) salvage GC- and GP-chemotherapy, (iii) consolidative radiotherapy, and (iv) salvage radiotherapy. The ureteral tumor and obturator LN, common iliac LN and para-aortic LN #1, and para-aortic LN #2 were cured by (i)–(iii), (ii)-(iii), and (ii)–(iv), respectively. These therapies may therefore be independently effective for heterogeneous cancers with different characteristics in different lymph nodes.

According to the UUT-UC collaboration's report, RNU with LND after neoadjuvant chemotherapy can accomplish pathological N0 in 50% of UTUC patients with nodal involvement [8], which leads to better overall survival (OS). Therefore, in the present case, we attempted neoadjuvant chemotherapy and curative LND based on possible anatomical lymphatic drainage from the lower ureter: obturator LNs (Figures 1(a) and 1(e)), a part of left common iliac LNs (Figures 1(b) and 1(f)), and internal and external iliac LNs (data not shown) [5, 6]. The metastasized obturator LN may have been cured only by LND with neoadjuvant GC-chemotherapy because the area was not irradiated (Figures 1(i), 1(m), and 1(q)). This result supports the UUT-UC collaboration's report mentioned above, and we are convinced that complete resection after neoadjuvant chemotherapy can cure metastatic UTUC.

However, the unremoved common iliac LN and para-aortic LNs increased in size early after the surgery. Matin et al. have reported that approximately 30% of LN involvement in lower UUT-UC unexpectedly spread to the para-aortic region [9], suggesting that the extent of LND in this case was insufficient. Further extended LND may be considered when conducting RNU as the common iliac LN was swollen.

Using data of bladder cancer, methods of combination chemotherapies are applied for UUT-UC because of its rarity [10]. As first line treatment, platinum-based combination chemotherapies including GC are generally used for advanced UC [10]. As second line treatment, GP-chemotherapy achieves an overall response rate of 30 to 70% [7]. In cisplatin-ineligible UC, taxanes with gemcitabine may have similar additional effects compared to carboplatin with gemcitabine (53% versus 45%) [11]. GC with paclitaxel may improve OS without increasing side effects [12]. Moreover, regimens with taxanes had a tolerable toxicity profile, even if patients have renal dysfunction post-RNU. In fact, complete response was obtained by treatment with GP [13]. In our case, the sizes of the two LNs (common iliac (Figures 1(r) and 1(v)) and para-aortic LN #1 (Figures 1(s) and 1(w)), resp.), with resistance to platinum-based chemotherapy, were also significantly reduced by GP-chemotherapy as a second-line treatment. Further compilation of cases cured by GP is needed to substantiate this superior UUT-UC survival outcome.

In patients with UUT-UC, bladder recurrence and distant metastases are the most frequently reported patterns, whereas isolated lymph node recurrence(s) is rare [14]. According to previous reports, adjuvant radiotherapy does not control lymph node metastases or improve OS [15–17]. However, Fan et al. showed that the group of UUT-UC patients treated with a prescribed dose of over 50 Gy as salvage radiotherapy had significantly longer progression-free survival and OS than the group of patients treated with a low dose; they emphasized that a high prescribed dose (≥50 Gy) was required in order to achieve curative outcomes [18]. Meanwhile, a small cohort study about consolidative radiotherapy reported the long-term clinical benefit of consolidative radiotherapy after chemotherapy (the prescribed dose ranged from 50 to 60 Gy) for nodal recurrences of UUT-UC treated with RNU [19]. In our case, the size of the para-aortic LN #2 that showed chemoresistance may have been significantly reduced by the total dose of 60 Gy as salvage radiotherapy (Figures 1(x), 1(ab), and 2). Additionally, both the common iliac LN and para-aortic LN #1 were also irradiated with consolidative radiotherapy after chemotherapy and are still reducing in size (Figures 1(v) and 1(w), 1(z) and 1(aa)). As a result, the patient has shown no evidence of recurrence and metastasis or any severe complications associated with radiotherapy for approximately 4 years from the initial diagnosis and for 18 months after radiotherapy.

## 4. Conclusions

Radiotherapy for LN metastasis in advanced UUT-UC has not yet been recommended. Our case suggests that salvage or consolidative radiotherapy combined with LND and perioperative chemotherapies may have clinical benefits in selected cases of advanced UUT-UC with LN metastasis. Further compilation of cases is needed to select appropriate patients, as well as standardizing radiotherapy methods, including range and dosage.

## Abbreviations

UUT-UC: Upper urinary tract urothelial carcinoma
LN: Lymph node
RNU: Radical nephroureterectomy
RT: Radiotherapy
CT: Computed tomography
UC: Urothelial carcinoma
GC-chemotherapy: Gemcitabine and cisplatin chemotherapy
LND: Lymph node dissection
GP-chemotherapy: Gemcitabine and paclitaxel chemotherapy
GTV: Gross tumor volume
CTV: Clinical target volume
RF: Radiation field.

## Supplementary Materials

Supplementary 1 Figure S1:* magnetic resonance imaging of the lower left ureteral tumor*. The ureteral tumor was 21 mm in diameter at the initial diagnosis (A). The tumor temporarily reduced in size after 3 courses of GC-chemotherapy (B). The tumor reenlarged after an additional 3 courses of GC-chemotherapy (C). The asterisks and red and light blue arrowheads indicate the bilateral femoral heads, ureteral tumor, and hydroureter, respectively.

Supplementary 2 Figure S2:* CT imaging of the lymph nodes*. Total CT imaging of the lymph nodes: obturator, common iliac, and para-aortic lymph nodes. Repetitive CT imaging was performed 19 times since initial diagnosis, to evaluate recurrence and metastasis. The white arrowheads indicate the lymph nodes.

Supplementary 3 Table S1: lymph nodes of clinical course of the case.
