# Supplementary material for: Clinical Impact of Consolidative and Salvage Radiotherapy for Lymph Node Metastasis in Upper Urinary Tract Urothelial Carcinoma
Source: Case Rep Urol. 2018 Apr 22;2018:1471839. doi: 10.1155/2018/1471839 (PMC5937622; doi:10.1155/2018/1471839)
Supplement: Supplementary 1 — Figure S1: magnetic resonance imaging of the lower left ureteral tumor. The ureteral tumor was 21 mm in diameter at the initial diagnosis (A). The tumor temporarily reduced in size after 3 courses of GC-chemotherapy (B). The tumor reenlarged after an additional 3 courses of GC-chemotherapy (C). The asterisks and red and light blue arrowheads indicate the bilateral femoral heads, ureteral tumor, and hydroureter, respectively. [file 1471839.f1.pdf]

Supplementary Figure 1

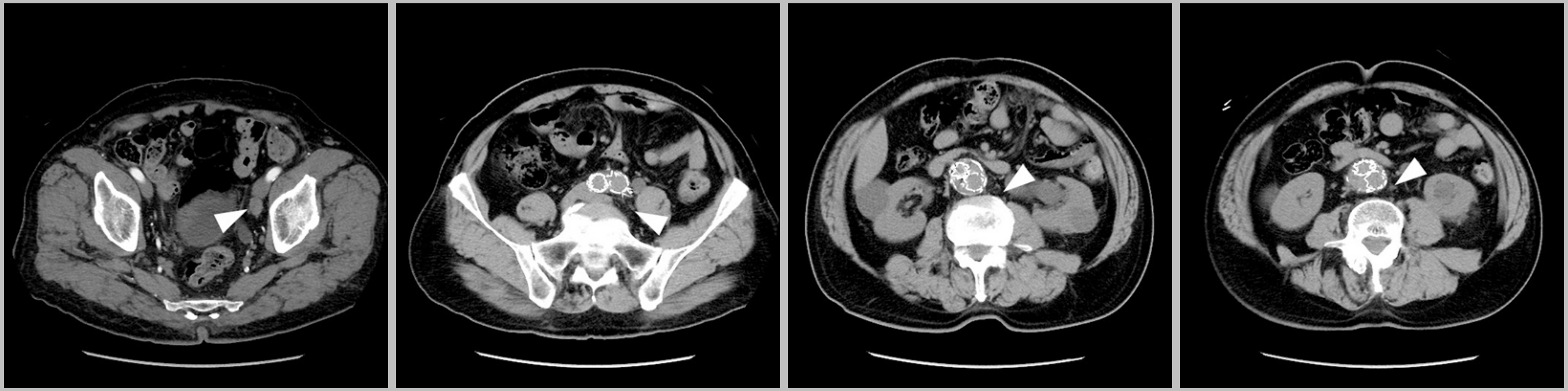

CT #1 obturator | CT #1, common iliac | CT #1, para-aorta #1 | CT #1, para-aorta #2

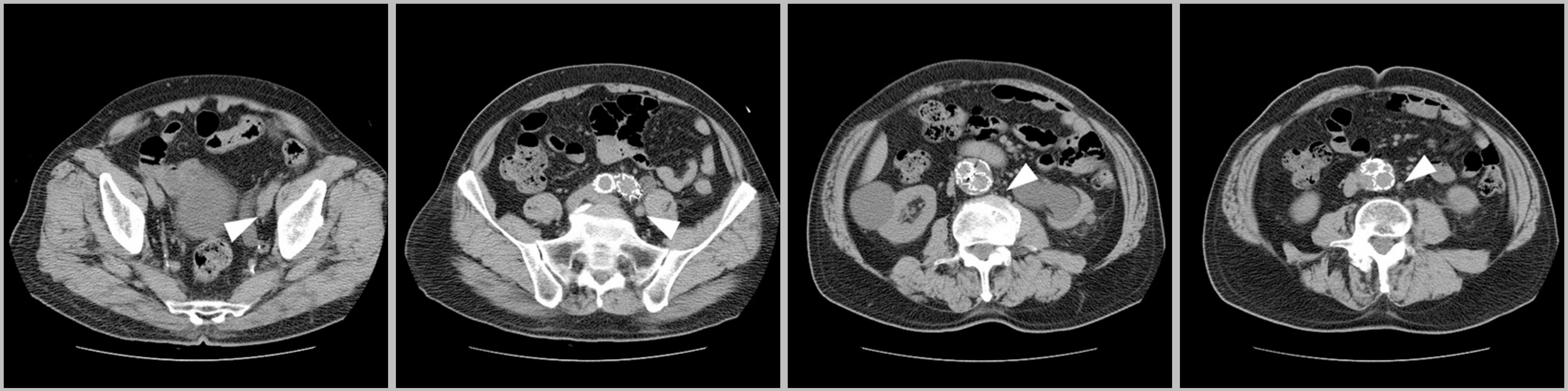

CT #2 obturator | CT #2, common iliac | CT #2, para-aorta #1 | CT #2, para-aorta #2

Supplementary Figure 1

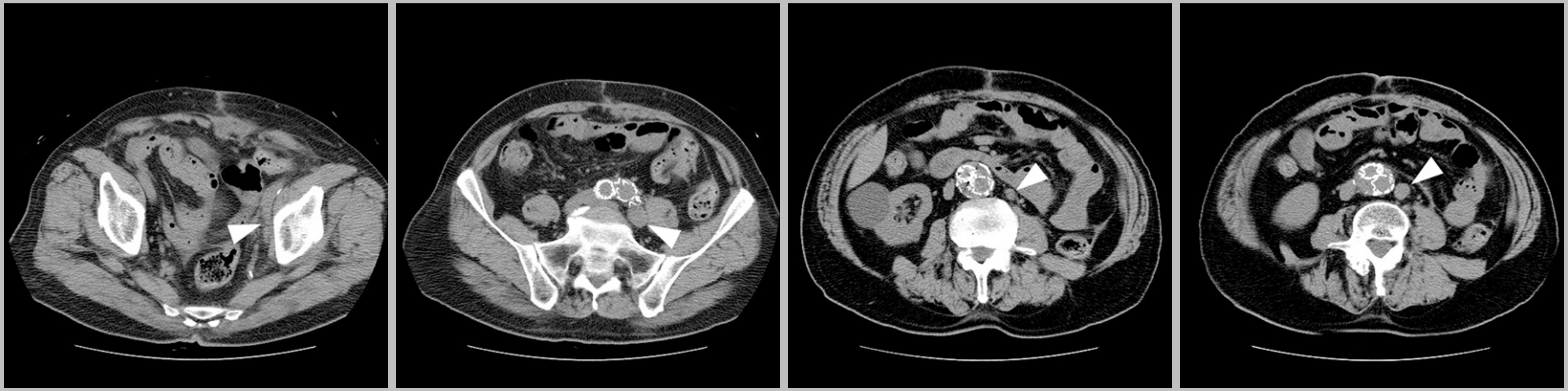

CT #3 obturator | CT #3, common iliac | CT #3, para-aorta #1 | CT #3, para-aorta #2

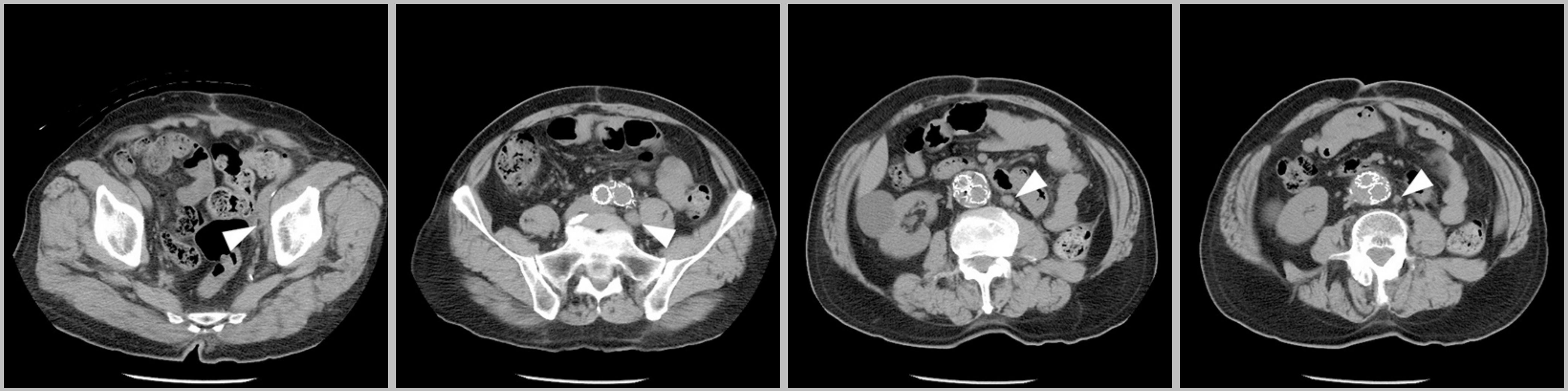

CT #4 obturator | CT #4, common iliac | CT #4, para-aorta #1 | CT #4, para-aorta #2

Supplementary Figure 1

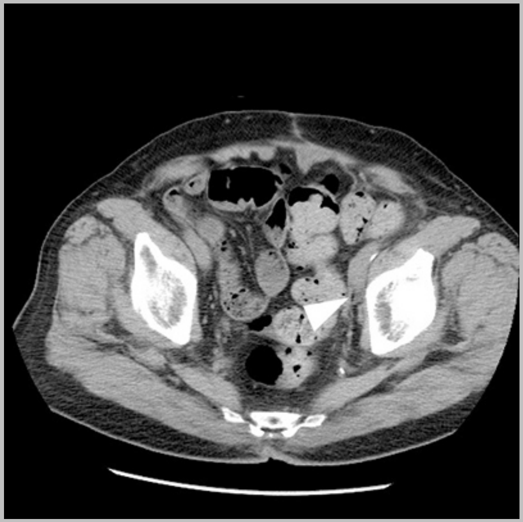

CT #5 obturator

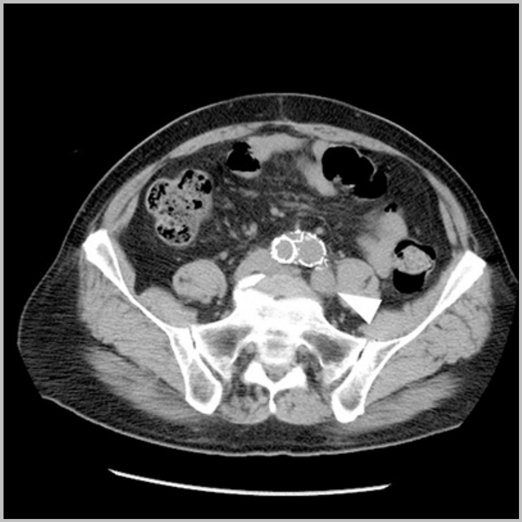

CT #5, common iliac

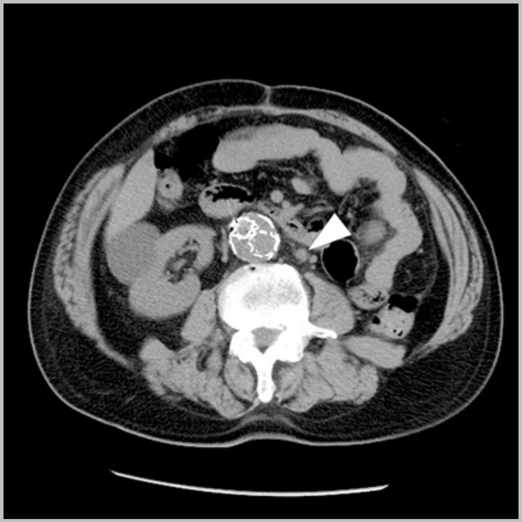

CT #5, para-aorta #1

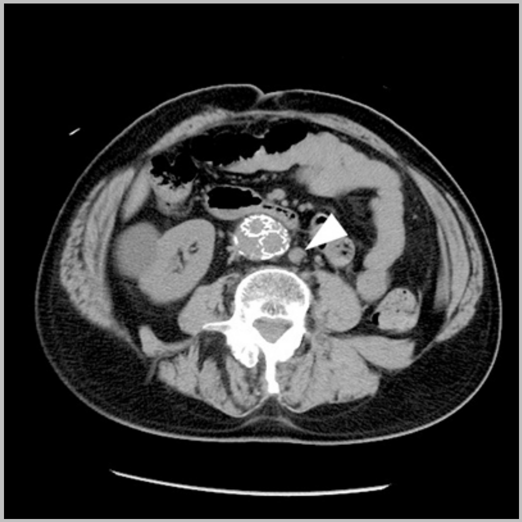

CT #5, para-aorta #2

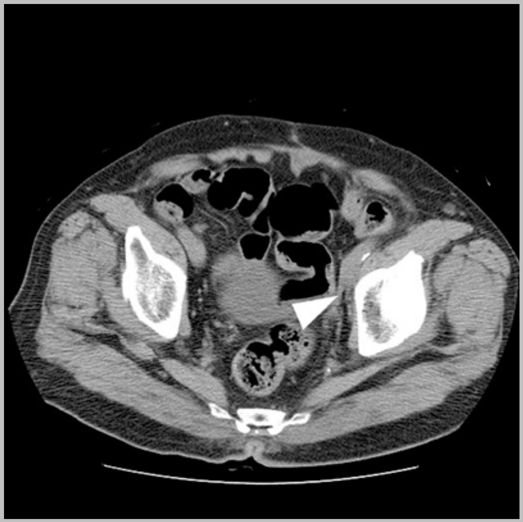

CT #6 obturator

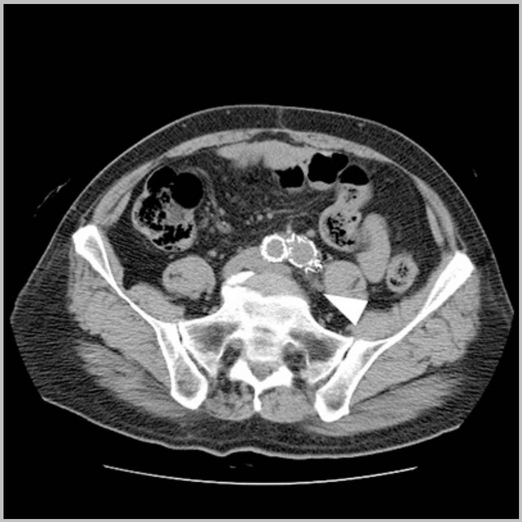

CT #6, common iliac

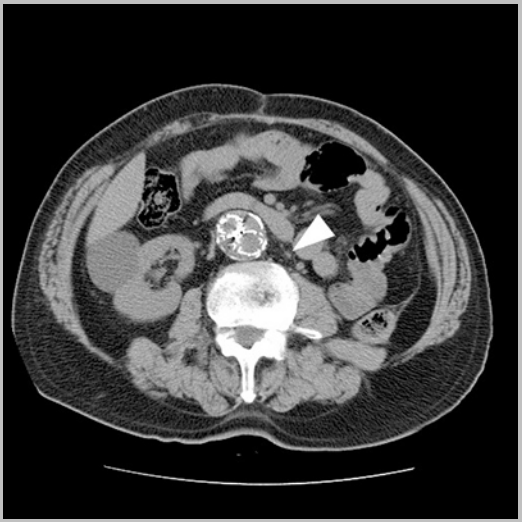

CT #6, para-aorta #1

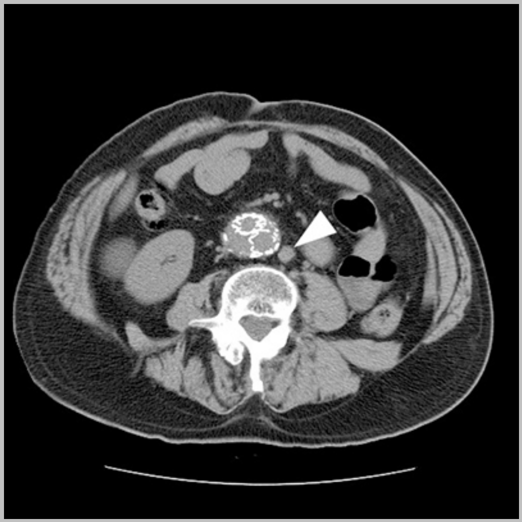

CT #6, para-aorta #2

Supplementary Figure 1

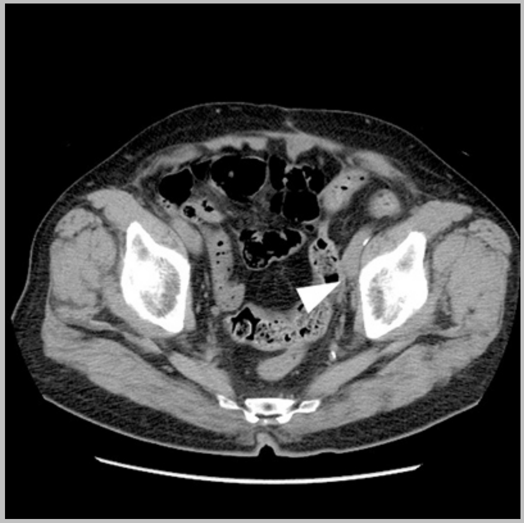

CT #7 obturator

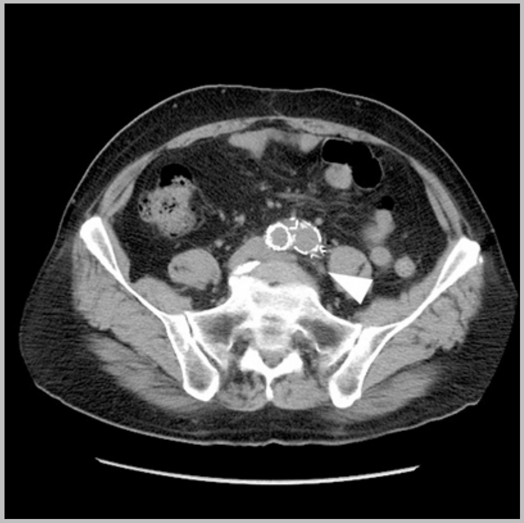

CT #7, common iliac

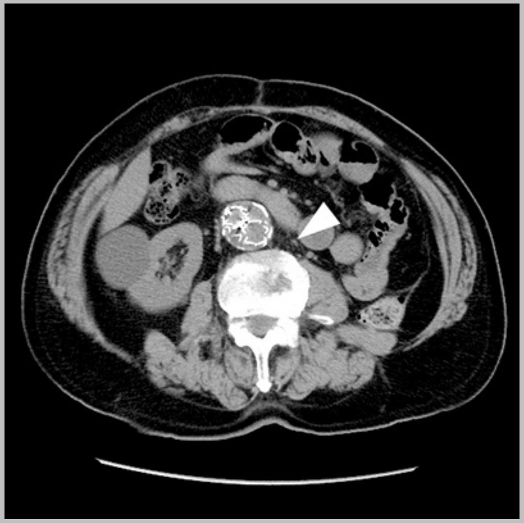

CT #7, para-aorta #1

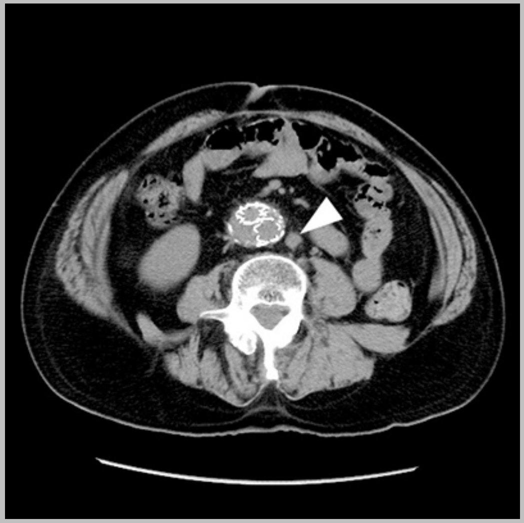

CT #7, para-aorta #2

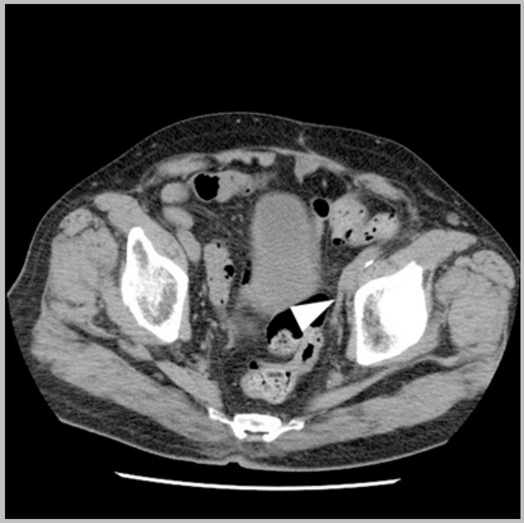

CT #8 obturator

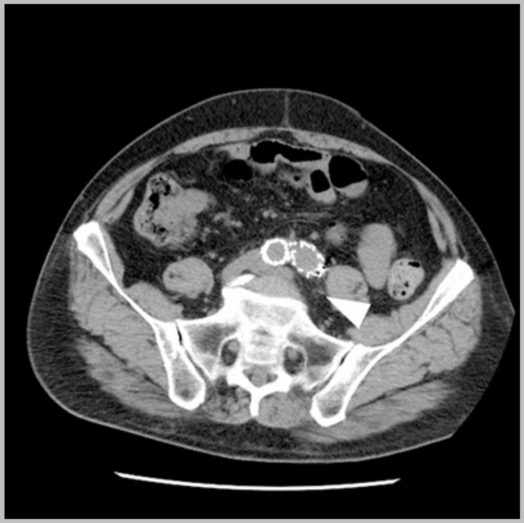

CT #8, common iliac

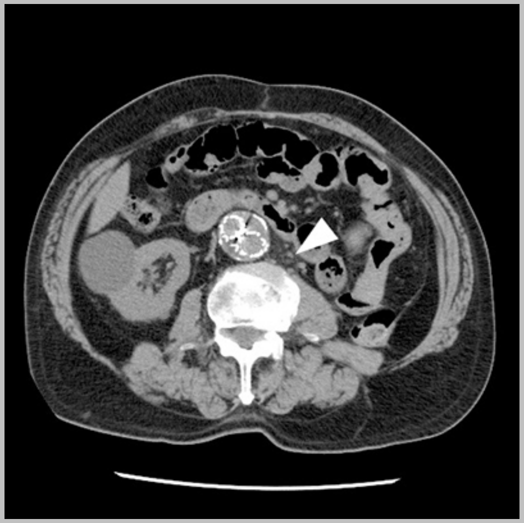

CT #8, para-aorta #1

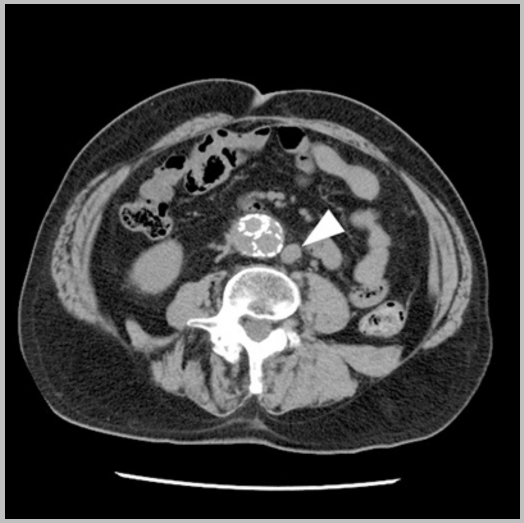

CT #8, para-aorta #2

Supplementary Figure 1

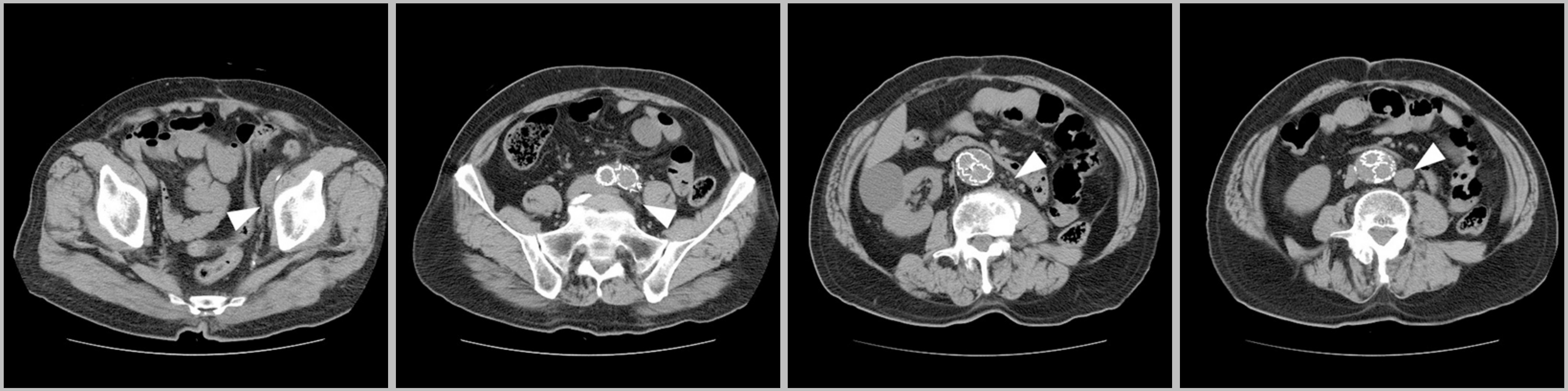

CT #9 obturator | CT #9, common iliac | CT #9, para-aorta #1 | CT #9, para-aorta #2

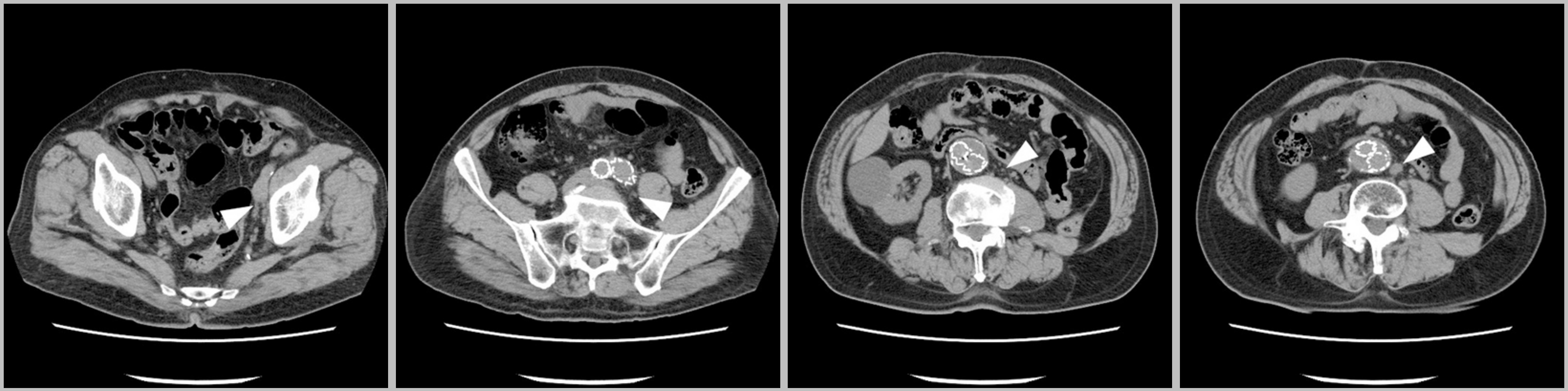

CT #10 obturator | CT #10, common iliac | CT #10, para-aorta #1 | CT #10, para-aorta #2

Supplementary Figure 1

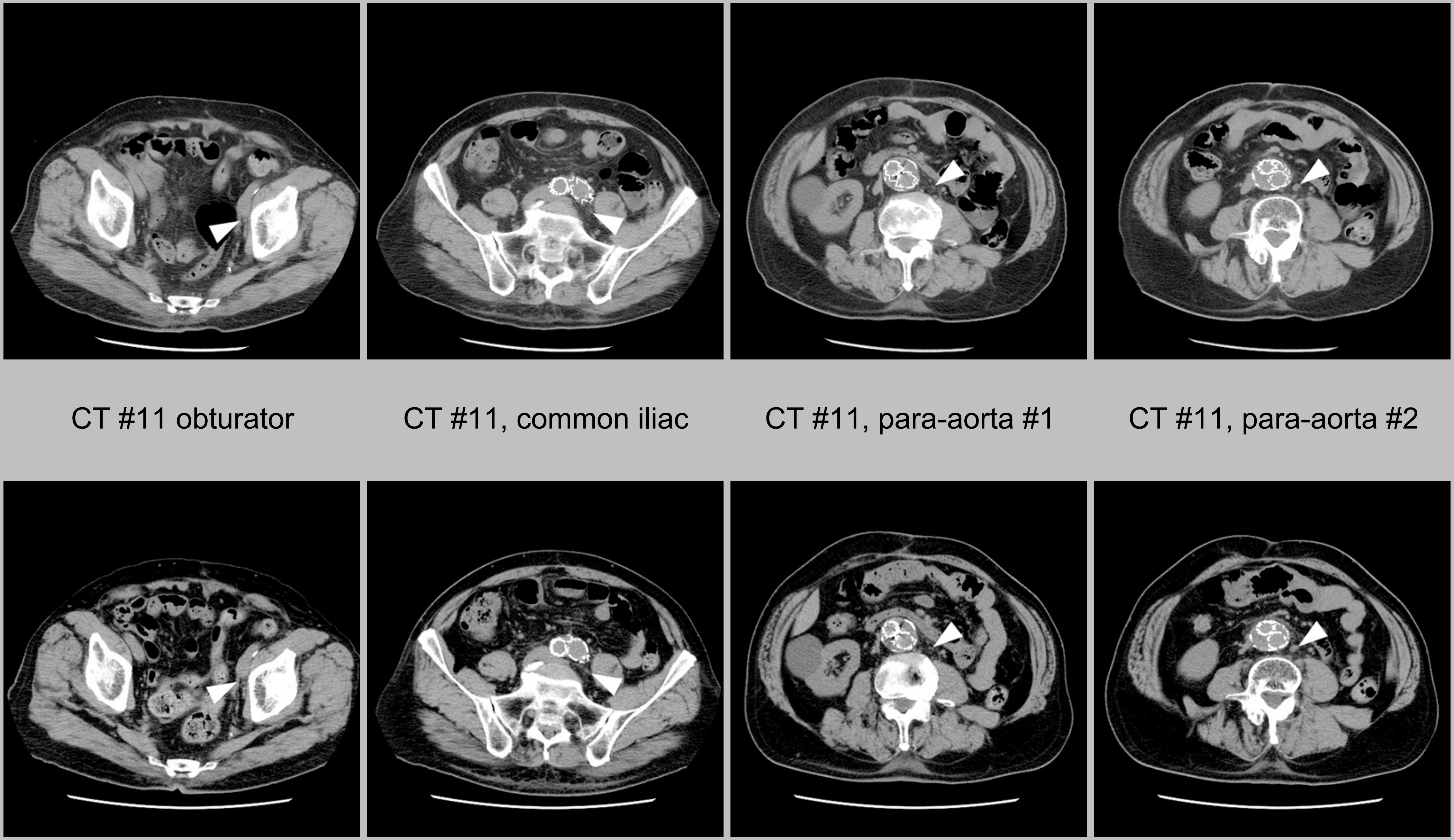

CT #11 obturator | CT #11, common iliac | CT #11, para-aorta #1 | CT #11, para-aorta #2

CT #12 obturator | CT #12, common iliac | CT #12, para-aorta #1 | CT #12, para-aorta #2

Supplementary Figure 1

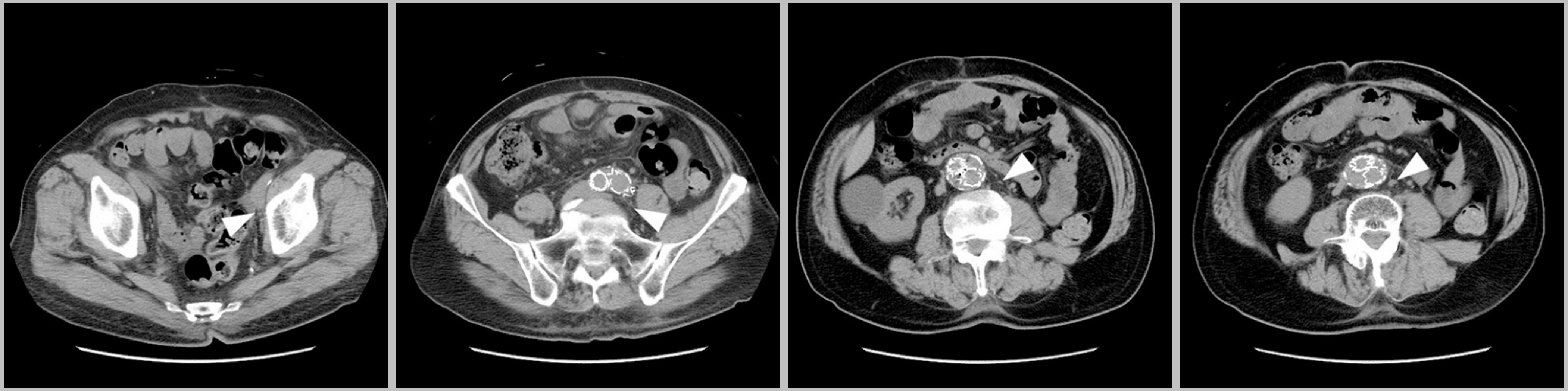

CT #13 obturator | CT #13, common iliac | CT #13, para-aorta #1 | CT #13, para-aorta #2

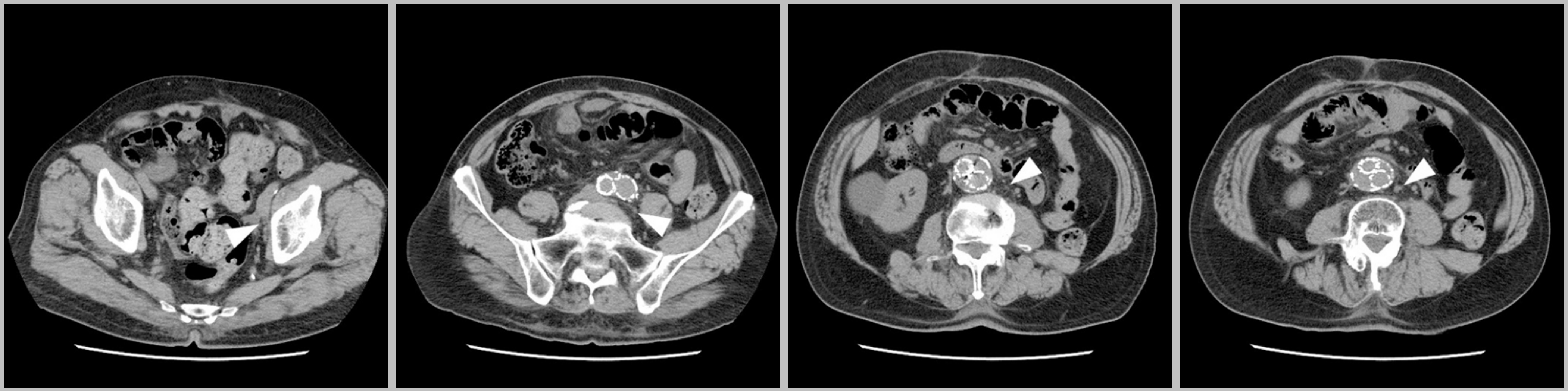

CT #14 obturator | CT #14, common iliac | CT #14, para-aorta #1 | CT #14, para-aorta #2

Supplementary Figure 1

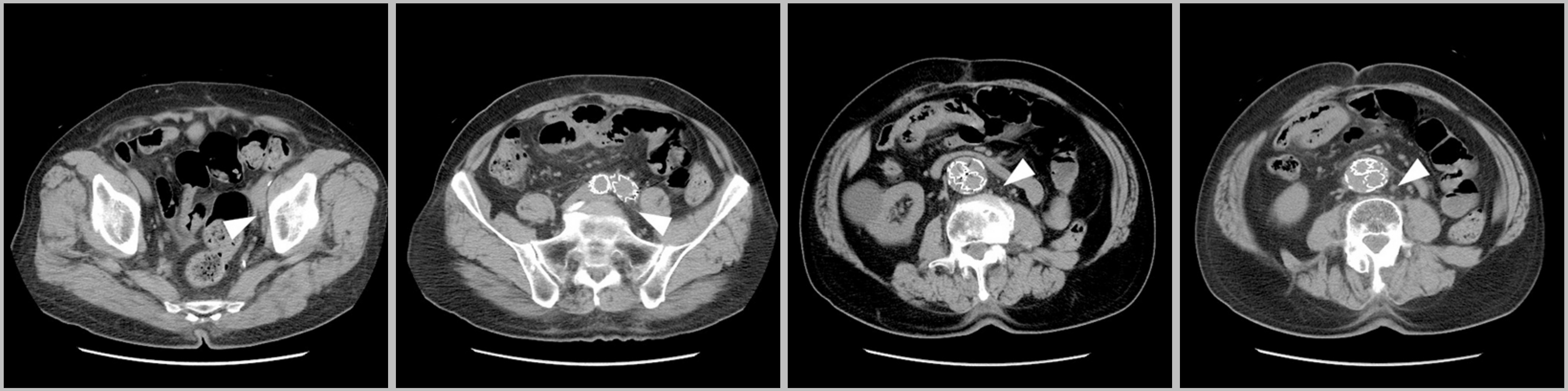

CT #15 obturator | CT #15, common iliac | CT #15, para-aorta #1 | CT #15, para-aorta #2

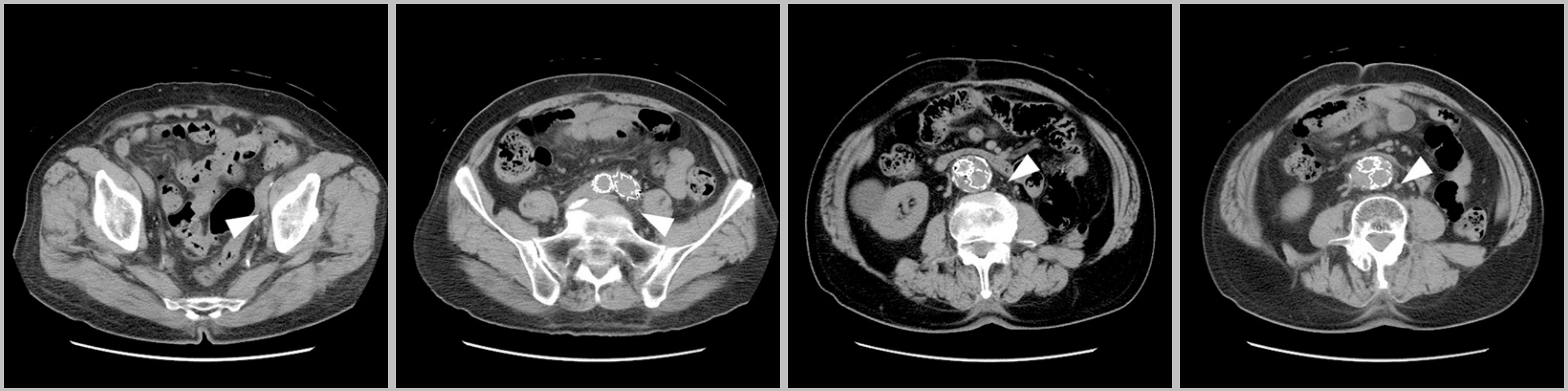

CT #16 obturator | CT #16, common iliac | CT #16, para-aorta #1 | CT #16, para-aorta #2

Supplementary Figure 1

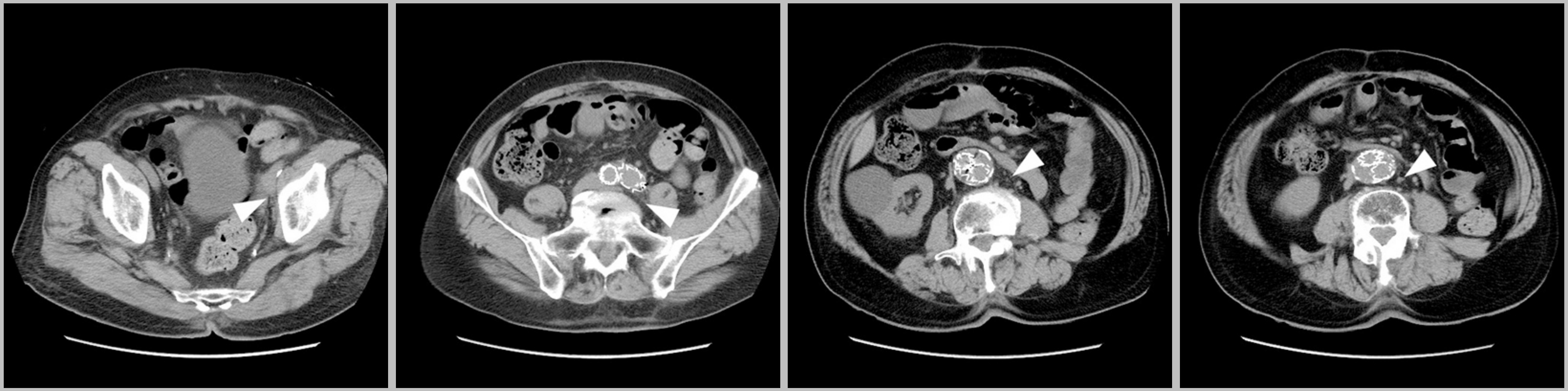

CT #17 obturator | CT #17, common iliac | CT #17, para-aorta #1 | CT #17, para-aorta #2

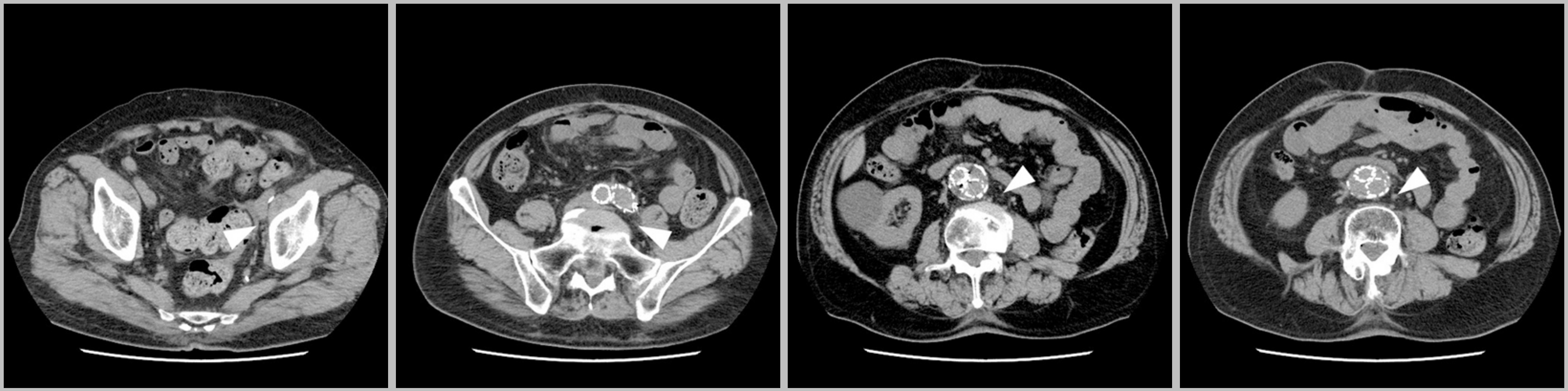

CT #18 obturator | CT #18, common iliac | CT #18, para-aorta #1 | CT #18, para-aorta #2

Supplementary Figure 1

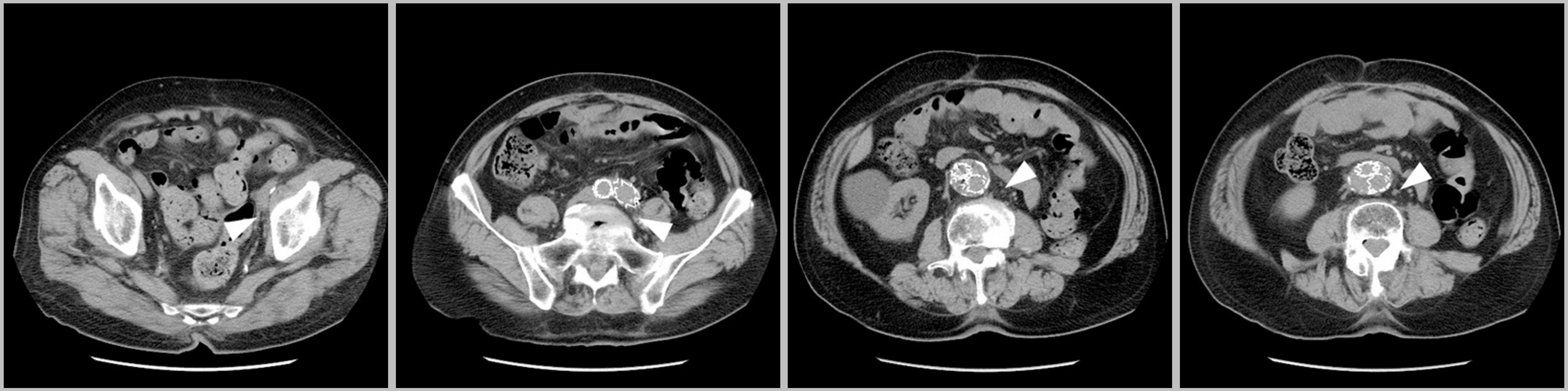

CT #19 obturator

CT #19, common iliac

CT #19, para-aorta #1

CT #19, para-aorta #2
