# Supplementary figures and images for: Clinical Impact of Consolidative and Salvage Radiotherapy for Lymph Node Metastasis in Upper Urinary Tract Urothelial Carcinoma
Source: Case Rep Urol. 2018 Apr 22;2018:1471839. doi: 10.1155/2018/1471839 (PMC5937622; doi:10.1155/2018/1471839)

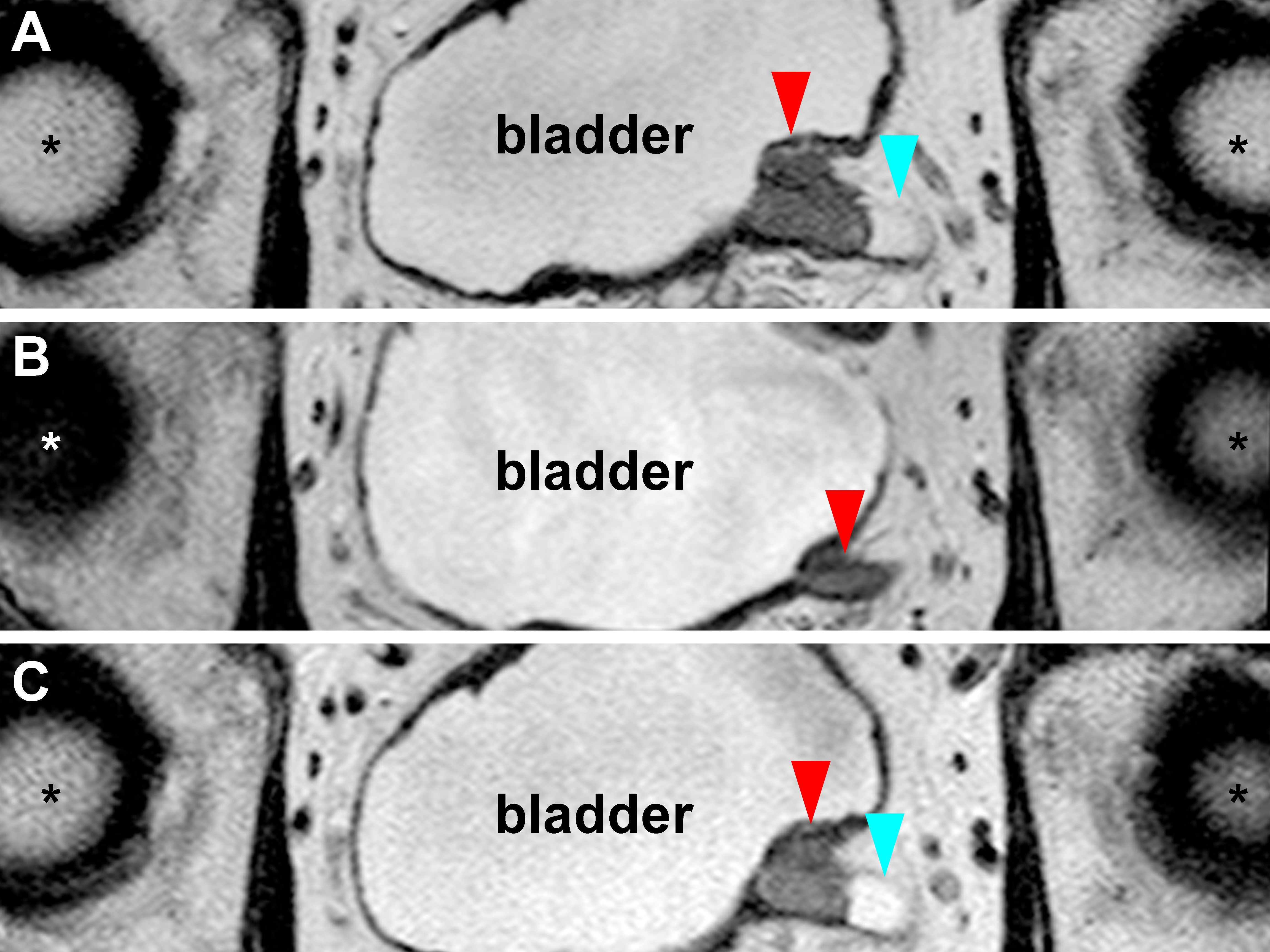

Supplement: Supplementary 2 — Figure S2: CT imaging of the lymph nodes. Total CT imaging of the lymph nodes: obturator, common iliac, and para-aortic lymph nodes. Repetitive CT imaging was performed 19 times since initial diagnosis, to evaluate recurrence and metastasis. The white arrowheads indicate the lymph nodes. [file 1471839.f2.jpg]
